# Time Course and Cellular Localization of SARS-CoV Nucleoprotein and RNA in Lungs from Fatal Cases of SARS

**DOI:** 10.1371/journal.pmed.0030027

**Published:** 2006-01-03

**Authors:** John M Nicholls, Jagdish Butany, Leo L. M Poon, Kwok H Chan, Swan Lip Beh, Susan Poutanen, J. S. Malik Peiris, Maria Wong

**Affiliations:** 1Department of Pathology, The University of Hong Kong, Pok Fu Lam, Hong Kong SAR, China; **2**Department of Pathology, University of Toronto, Toronto General Hospital, Toronto, Ontario, Canada; **3**Department of Microbiology, The University of Hong Kong, Pok Fu Lam, Hong Kong SAR, China; **4**Department of Microbiology, Toronto Medical Laboratories and Mount Sinai Hospital, University of Toronto, Toronto, Ontario, Canada; Centers for Disease ControlUnited States of America

## Abstract

**Background:**

Cellular localization of severe acute respiratory syndrome coronavirus (SARS-CoV) in the lungs of patients with SARS is important in confirming the etiological association of the virus with disease as well as in understanding the pathogenesis of the disease. To our knowledge, there have been no comprehensive studies investigating viral infection at the cellular level in humans.

**Methods and Findings:**

We collected the largest series of fatal cases of SARS with autopsy material to date by merging the pathological material from two regions involved in the 2003 worldwide SARS outbreak in Hong Kong, China, and Toronto, Canada. We developed a monoclonal antibody against the SARS-CoV nucleoprotein and used it together with in situ hybridization (ISH) to analyze the autopsy lung tissues of 32 patients with SARS from Hong Kong and Toronto. We compared the results of these assays with the pulmonary pathologies and the clinical course of illness for each patient. SARS-CoV nucleoprotein and RNA were detected by immunohistochemistry and ISH, respectively, primarily in alveolar pneumocytes and, less frequently, in macrophages. Such localization was detected in four of the seven patients who died within two weeks of illness onset, and in none of the 25 patients who died later than two weeks after symptom onset.

**Conclusions:**

The pulmonary alveolar epithelium is the chief target of SARS-CoV, with macrophages infected subsequently. Viral replication appears to be limited to the first two weeks after symptom onset, with little evidence of continued widespread replication after this period. If antiviral therapy is considered for future treatment, it should be focused on this two-week period of acute clinical disease.

## Abstract 2

The SARS coronavirus targets primarily the pulmonary alveolar epithelium. Viral replication seems limited to the first two weeks after symptom onset and restricted to the lungs.

## Introduction

Severe acute respiratory syndrome (SARS) is a new disease that originated in the Guangdong province of China in November 2002, and subsequently spread to Hong Kong in February 2003 [1,2]. Facilitated by busy international traffic, it soon caused major disease outbreaks in multiple cities around the world over the following five months [3,4]. It had an alarming tendency to spread to health care workers, and unlike most respiratory viral infections, predominantly involved the lower respiratory tract. The disease was associated with an unusually high morbidity and mortality; 20%–30% of patients developed severe respiratory symptoms requiring intensive care support [2,3,5]. Worldwide, the average case mortality rate was 10.0%, but in Hong Kong and Toronto, the case mortality rate was 17.0%, with older patients and those with chronic illnesses having a worse outcome [1,2,5].

The causative agent has now been determined to be a novel coronavirus (SARS-CoV) that is genetically distinct from any previously identified coronavirus known to cause disease in animals or humans [1,6–8]. In a primate model system, experimental infection of cynomolgus macaques with SARS-CoV alone was sufficient to cause a disease complex similar to that observed in humans [9,10]. Genetically, the coronavirus species closest to SARS-CoV are those isolated from small animals traded for culinary purposes, such as the Himalayan palm civet and raccoon-dog, which show 99.8% sequence homology but differ by the insertion of a 29-nucleotide fragment [11]. It is postulated that both coronaviruses have probably arisen from a common natural reservoir, with facilitated interspecies transmission and possibly genetic alteration to SARS-CoV during the close handling and trading of the animals in the same environment.

The pulmonary pathology observed in limited postmortem material has been reported together with the clinical symptomatology of SARS [2,8,12–15]. The detection of the coronavirus in relation to pulmonary changes is a critical factor in the understanding of the pathogenesis of the disease. However, this has been hampered by the lack of readily available monoclonal anti-SARS antibodies, as well as the impracticability of extensive electron microscopic examination. Furthermore, although investigation of the time course of SARS-CoV infection with respect to the detection of SARS-CoV RNA has been done by PCR methods, there have been no systematic, large-scale investigations of the temporal or cellular detection of SARS-CoV in lung tissues. Additionally, the extent of organ distribution has not been examined in great detail. Indeed, a recent single-case publication on the immunohistochemical detection of SARS highlighted “…the need for more cases of SARS to be studied to determine the temporal relationship between the duration of illness and viral clearance in human lung tissue…” [15].

This study therefore had three purposes. The first was to investigate the presence of SARS-CoV by immunohistochemistry (IHC) and in situ hybridization (ISH) in lung autopsy specimens from patients who died at different time points of the disease. The second purpose was to determine the cellular distribution of SARS within the lung, and the third was to examine extrapulmonary tissues of patients who died of SARS to determine the extent of systemic distribution.

## Methods

### Patient Profiles and Material

A total of 32 patients who died with SARS-CoV infection confirmed by PCR, serological, or viral culture tests were included in the study. The patients came from two geographical regions affected by SARS (Hong Kong [HK] and Toronto, Canada [TO]). For HK cases 1 and 10–13, whole lung specimens were perfused at autopsy with 4% neutral buffered formalin at 20–30 cm H_2_O pressure until the lungs were fully expanded, before immersion in formalin fixative. Multiple blocks were extensively sampled from all lobes of the lungs for HK cases 1, 6, 7, and 9–13; limited representative blocks were sampled from HK cases 2–5 and 8. For all Toronto cases, approximately half of each lung lobe was removed at autopsy, and a portion was snap-frozen for molecular studies. Multiple blocks of tissue were processed from each lobe for light microscopic examination. Stains for microorganisms and for collagen, and immunohistochemical stains for microorganisms, were performed as necessary. The clinical profiles were retrieved from the patients' records. HK case 1 was a patient who died in the quarantine period for SARS exposure during hospitalization for congestive heart failure. PCR of a throat swab was positive on the fifth day after symptom onset, and she developed fever and respiratory symptoms thereafter. HK case 13 was a patient who died unexpectedly during convalescence from SARS. All other patients died of SARS with progressive deterioration in lung function. All HK patients except cases 1 and 13 had received ventilatory support during life. The pulmonary histopathological features of six of the HK cases (2–6 and 8) have been previously reported [12]. In addition to the autopsy tissue from all HK cases, HK case 5 also had an open lung biopsy, which was also included in this study. Descriptions of the procurement and clinical courses of the TO cases have been described in previous reports [14,16].

### Development of a Monoclonal Antibody against SARS-CoV Nucleoprotein

BALB/c mice were immunized intraperitoneally with 0.1 ml of heat-killed SARS-CoV HKU39849-infected FRhK4 cell lysate (10^7^ TCID50/ml). Injections of similar doses were repeated biweekly for 2 mo. Four days after the last booster, 10^8^ spleen cells from an immunized mouse were fused with 10^7^ of NSI myeloma cells with polyethylene glycol (PEG, molecular weight 4,000; BDH, Poole, United Kingdom) as the fusing agent. Hybridomas were screened for production of antibodies against SARS-CoV HKU39849-infected cells and recombinant SARS-CoV nucleocapsid protein by ELISA. Those that produced SARS-CoV nucleoprotein-specific antibodies were cloned twice by limiting dilution. Purified hybridomas were then injected intraperitoneally into mineral oil-primed mice for the production of ascitic fluid. Monoclonal antibodies (4D11 and 3E4) were purified from the ascitic fluid by precipitation with 50% ammonium sulfate and subcloned to ensure monoclonality.

### Development of Transfection Controls

The open reading frames of spike (S), envelope (E), and N genes of SARS-CoV were cloned into the AgeI site of a protein expression vector, pcDNA3A [17]. To express the above viral proteins in eukaryotic cells, these protein expression plasmids were transfected into 293T cells individually or in appropriate combinations. Briefly, 1 μg of each of the plasmids was transfected into 293T cells by Lipofectamine 2000 (Invitrogen, Carlsbad, California, United States) as instructed by the manufacturer. At the indicated posttransfection time points, transfected cells were harvested and washed with PBS, then centrifuged at 1,600 rpm for 10 min. Pelleted cells were fixed in PBS with 0.1% glutaraldehyde and 2% formaldehyde, and stored at 4 °C. SARS-CoV S and E gene-transfected cells were used as negative controls, and SARS-CoV N gene-transfected cells were used as positive controls for IHC to confirm the specificity of the monoclonal antibodies against SARS-CoV nucleoprotein.

### Immunohistochemistry

The primary antibodies used included those against cytokeratin (1:50, clone AE1/AE3 [Dako, Glostrup, Denmark]), CD68 (1:50; clone KP1, Dako), EMA (clone E29, Dako), thyroid transcription factor 1 (TTF1; clone 8G7G3/1, Zymed/Invitrogen), chromogranin (clone LK2H10 [Ventana, Tucson, Arizona, United States]), DC-SIGN (a gift from Professor J-L Virilizier, Pasteur Institute, Paris, France), LCA (clone T29/33, Dako), and SARS-CoV N (as described above) (1:400, clone 4D11). Antigen retrieval was performed by microwaving sections in 10 mM citrate buffer (pH 6.0) for 15 min and incubating with 1:200 4D11 antibody at 4 °C overnight. Secondary labeling was performed with biotinylated rabbit anti-mouse (Dako #E-0354) at 1:100 for 30 min at room temperature and was followed by incubation with streptavidin-ABC complex (Dako #K-0377) at 1:100 for 30 min at room temperature and color development by the 3-amino-9-ethylcarbazole (AEC) substrate kit (Vector Laboratories, Burlingame, California, United States; #SK-4200) at room temperature (15–30 min). For double labeling of lung tissue sections, the 4D11 antibody was labeled with FITC, and a TRITC anti-mouse secondary antibody was used. Sections were microwaved in 10 mM citrate buffer (pH 6.0) for 15 min, blocked with 10% normal donkey serum for 10 min at room temperature, incubated with the non-SARS monoclonal overnight, then incubated with TRITC-conjugated donkey anti-mouse antibody at 1:100 for 1 h at room temperature. The FITC-conjugated 4D11 antibody was incubated at 1:100 for 1 h at room temperature, followed by counterstaining of the nuclei with DAPI for 4 min and mounting with DAKO fluorescence mount (Dako #S-3023). Examination was performed with a Nikon Eclipse E-800 fluorescent microscope with a dual FITC/rhodamine filter.

### In Situ Hybridization

We produced digoxigenin-labeled antisense riboprobes specific for the N, E, and S genes of SARS-CoV, and these were pooled and hybridized with 30 μg/ml proteinase K-treated lung sections for 30 min at room temperature. The hybridization buffer (250 μg/ml salmon sperm DNA, 125 μg/ml rat total RNA, 200 mg/ml yeast tRNA, 50% deionized formamide, 10% dextran sulfate, 1× Denhardt's solution, 1 mM EDTA [pH 8.0], 0.01% sodium pyrophosphate, 0.3 M sodium chloride, and 10 mM Tris-HCl [pH 8.0]) was mixed with the probes and hybridized overnight at 50°C. The hybridized probes were detected with mouse anti-digoxigenin at 1:100 in 10% normal rabbit serum for 1 h at room temperature, then biotinylated rabbit anti-mouse (Dako, E-0354) at 1/100 for 30 min. at room temperature, followed by detection with AEC substrate kit (Vector, SK-4200) at room temperature (up to 30 min). Prior to the tests, the sensitivity of the SARS-CoV probes were verified semiquantitatively by hybridization to separate cell block preparations of a SARS-CoV infected FRHK4 cell line harvested at 8 and 24 h.

### RT-PCR

SARS-CoV RT-PCR was completed on fresh (HK cases 1 and 10–13), fixed (HK cases 2–9), and snap-frozen (all TO cases) lung tissues from all patients. For the HK cases, total RNA was extracted by standard methods and reverse transcribed to cDNA with reverse transcriptase. PCR was performed with initial denaturation at 94 °C for 8 min followed by 40 cycles of 94 °C for 1 min, 50 °C for 1 min, and 72 °C for 1 min using primers as previously described [1,5,12]. For the TO cases, RT-PCR was completed using RealArt HPA-Coronavirus LC-RT assay (Artus, Hamburg, Germany) as previously described [16,18].

## Results

The HK and TO cases' clinical profile, treatment history, and SARS-CoV IHC, ISH, and RT-PCR results are summarized in Table 1.

**Table 1:**
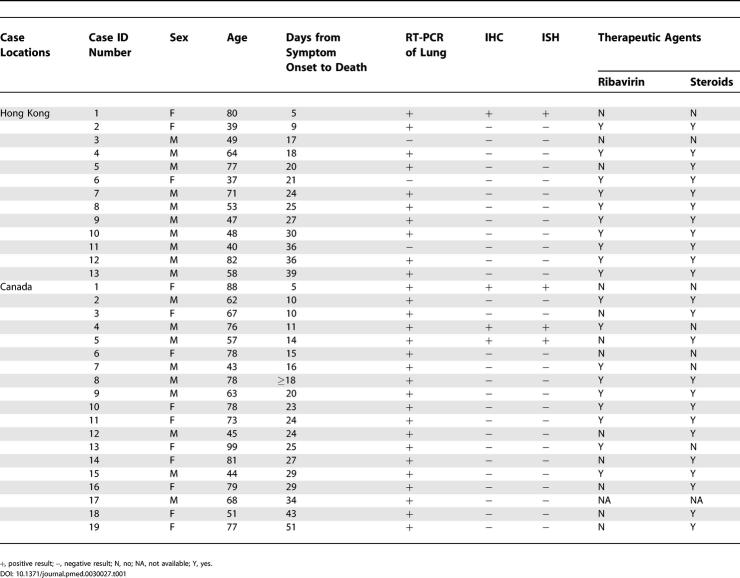
Profile of Cases Listed According to Duration of Disease from Symptom Onset for Each Location

### Lung Pathology

HK patient 1, who died 5 d after symptom onset during quarantine for SARS contact, showed moderate interstitial and alveolar edema with occasional epithelial desquamation and regeneration. A moderate number of macrophages with abundant foamy cytoplasm had accumulated in the alveolar spaces, but giant cells were not observed. In addition, features of aspiration pneumonia were present, involving small bronchi and adjacent parenchyma in all lobes of the lung. HK patients 2–12 showed established changes indicating diffuse alveolar damage (DAD) at different phases of disease progression that affected the lungs to varying extents. Lung pathology of the TO cases has been described previously [14].

Although most of the advanced histological changes revealing DAD can be attributed to ventilation, it should be pointed out that there were also changes of DAD present in five of the TO cases in which no intubation was carried out; thus, viral damage is therefore the most likely mechanism of these changes.

### SARS-CoV IHC, ISH, and RT-PCR Results

The transfected cells showed strong cytoplasmic staining for transfectants containing the N gene with no staining on the M or S gene transfectants (Figure 1), confirming the specificity of the monoclonal antibody for the N protein. The control sections of FRhK4 cells showed cytoplasmic staining that was focal at 8 h and diffuse at 24 h.

**Figure 1:**
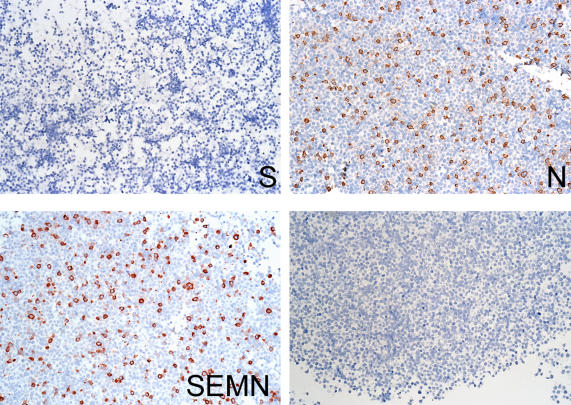
Confirmation of the Specificity of the Monoclonal Antibody 4D11 for the SARS-CoV Nucleocapsid Protein SARS-CoV immunohistochemical staining of 293T cells transfected with spike (S), nucleocapsid (N), and spike, nucleocapsid, membrane, and envelope (SEMN) genes of SARS-CoV. Untransfected control is shown in the lower right photomicrograph. The monoclonal antibody 4D11 identifies the N protein. AEC stain with hematoxylin counterstain; magnification 100×.

Four cases showed positive staining for N protein by IHC (HK case 1 and TO cases 1, 4, and 5) (Figure 2). All four of these cases died within 14 d of symptom onset. No staining of the bronchial epithelium was present (Figure 2A), and there was only focal staining of the bronchiolar epithelium (Figure 2B), which showed no evidence of necrosis or regeneration. This observation was confirmed by electron microscopy with viral particles identified in ciliated cells. There was cytoplasmic staining of flattened type 1 pneumocytes as well as alveolar macrophages (Figure 2C and 2D). Adjacent hilar lymph nodes were available for examination in one case (HK case 1), and, of six nodes sampled from this patient, only one isolated mononuclear cell was noted that stained positive for N protein but was negative for the N gene by ISH. Although the staining of the pneumocytes was diffusely cytoplasmic, the staining of the alveolar macrophages was coarsely granular (Figure 2F). For all patients who died beyond 14 d after symptom onset, only scattered single positive cells were seen in lung sections (Figure 2G and 2H), and these were in flattened pneumocytes as well as in mononuclear cells. In one of the Toronto cases (TO case 1) a thrombus was identified (Figure 2E), and positive elongated cells as well as rounded mononuclear cells were identified in this thrombus. This thrombus was present in only one section, and further labeling to confirm the etiology of the infected cells could not be performed. None of the multinucleated epithelial cells seen in any of the patients were positive by ISH or IHC.

**Figure 2:**
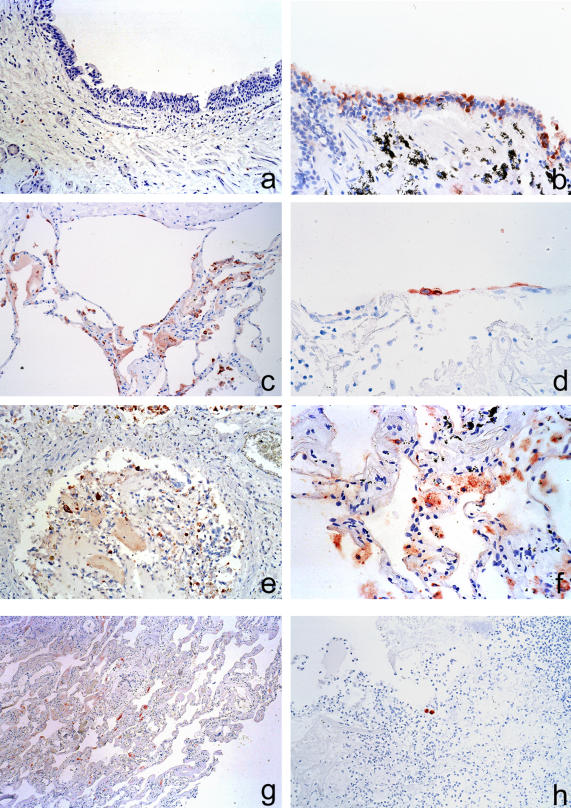
Examples of SARS-CoV IHC/ISH Results No staining for N protein is present in the bronchus of HK case 1 (A), but there is focal positive staining of bronchiolar epithelium in this case (B). Positive staining is seen in epithelial cells and detached cells in the alveoli in TO case 1 (C), which on higher magnification morphologically resemble type 1 pneumocytes (D). In TO case 2, there is a thrombus present which contains mononuclear and spindle-shaped cells (E). Carbon-containing macrophages are also positive (F). Ten days after symptom onset, positive staining was reduced and is noted mainly in the exudates (G) with only very focal positive mononuclear cells seen (H). AEC stain with hematoxylin counterstain; magnification 100×.

In the four cases that stained positive for N protein by IHC, double labeling with the directly conjugated FITC SARS monoclonal antibody and antibodies for macrophages and epithelial cells showed that the infected cells were positive for EMA (anti-epithelial) and CD68 (anti-macrophage) (Figure 3A and 3B). The number of infected cells was greatest in TO case 1 and HK case 1, both of whom died 5 d after symptom onset. Of interest, TO case 1 showed positive staining of epithelial cells but no staining of macrophages (Figure 3C and 3D). Macrophages showed positive coarse granular staining in HK case 1 and TO cases 4 and 5, who died 11 and 14 d after symptom onset, respectively (Figure 3E). No staining was seen in chromogranin-positive or DC-SIGN-positive cells, and no colocalization with TTF1 (a marker for type 2 pneumocytes) was seen. No staining of lymphocytes was identified. TO case 5, who died on the day 14 after symptom onset, had only scattered single positive cells, and these were in flattened epithelial cells and mononuclear cells.

**Figure 3:**
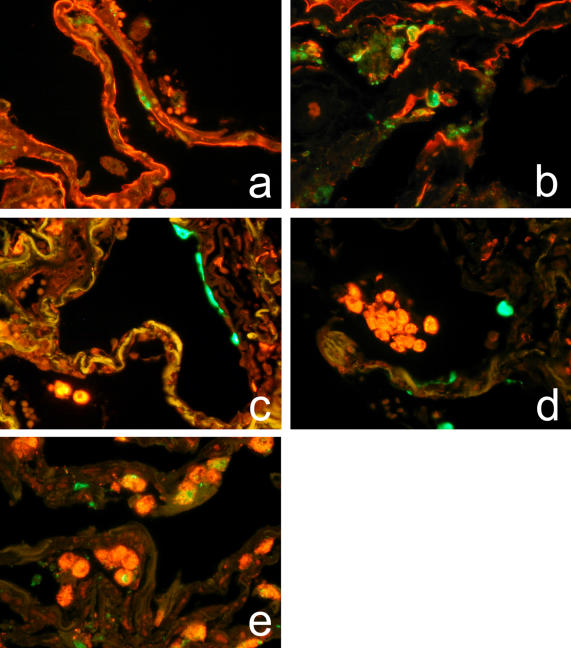
Colocalization of SARS-CoV Infection of Human Lung Tissues Shown are tissue samples stained with SARS-CoV monoclonal nucleocapsid FITC and epithelial membrane antigen TRITC. At day 5 postinfection, there is positive staining of type 1 pneumocytes (A), with epithelial cell debris present in the alveolar lumen (B). In one case (TO case 1), there is positive staining for SARS-CoV of the type 1 pneumocytes with no staining of CD68 positive macrophages (C and D). HK case 1, however, shows FITC staining within the cytoplasm of CD68-positive cells (E).

The same four cases that showed positive staining for N protein by IHC (HK case 1 and TO cases 1, 4, and 5) were also positive by ISH for the pooled S, E, and N SARS-CoV genes. Multiple lung samples from HK case 1 showed strong cytoplasmic signals in alveolar cells, including desquamating pneumocytes and alveolar macrophages. Weak signals were detected in occasional macrophages scattered along the bronchial epithelium. No signals were detected in endothelial or stromal cells. In addition to the four cases that stained positive for SARS-CoV by IHN and ISH, a few SARS-CoV infected cells were also detected by ISH and IHC in an open lung biopsy taken 10 d after symptom onset from HK case 5; however, both ISH and IHC were negative on the autopsy lung sample taken from this patient after death (20 d after symptom onset).

Results of RT-PCR are listed in Table 1. Autopsy lung samples from all but three HK cases were positive for SARS-CoV by RT-PCR. When IHC and ISH staining was compared with RT-PCR viral load and disease duration, positive cells by IHC and ISH were identified mainly in cases who had died less than 14 d from onset of illness and in whom lung tissues were associated with viral loads greater than 10^5^ copies per gram of tissue. In two of the cases that were positive for IHC in the lung, other organs were examined for IHC. In HK case 1, when there was strong positive IHC and ISH staining of the lung identified, no IHC- or ISH-positive cells were identified in other organs examined (adrenal gland, kidney, spleen, liver, and heart). In TO case 4, which also showed positive lung staining, no other organs were positive by IHC or ISH (liver, spleen, and kidney). Examination of three other TO cases that stained negative in the lung also showed no positive extrapulmonary staining.

## Discussion

SARS-CoV nucleocapsid protein and RNA were detected in lung autopsy samples from four of the seven patients who died within 2 wk after illness onset, all of whom had high (greater than 10^5^ copies per gram) RT-PCR viral loads in the autopsy lung tissue. SARS-CoV nucleocapsid protein and RNA were also detected in an open lung biopsy from a patient on day 5 after symptom onset (before death), but not in the same patient's autopsy lung specimen collected on day 20 after symptom onset. Among those lung samples staining positive by ISH or IHC, the signals localized mainly to the alveolar epithelial cells, with lower intensity in alveolar macrophages, indicating that the former are the chief target cells of the virus. Only scattered positive cells were identified in the bronchiolar epithelium, and no significant staining of the bronchial epithelium was observed. No distinctive spread to regional lymph nodes was seen, and we were not able to determine colocalization with DC-SIGN–positive cells. Using an oligonucleotide probe with signal amplification, Nakajima et al. demonstrated SARS-CoV genomes in the alveolar epithelium and macrophages of a 46-year-old woman with SARS, but details of the clinical course of the disease were not stated [19]. The preferential localization of the virus to alveolar cell components relative to the bronchial epithelium seen at the time of death suggests that the alveolar environment might be more permissive to viral replication, and could also account for the high incidence of lower respiratory tract involvement. It should be noted, however, that there are no studies of SARS in humans in which bronchial or tracheal biopsy material from nonfatal cases has been examined. Therefore, whether there is SARS-CoV replication in these parts of the respiratory tract, as is the case in nonhuman primates [10], remains unanswered.

Among the 25 patients who died later than two weeks after symptom onset, no ISH or IHC signals were detected in postmortem lung tissues, even in lesions that were morphologically earlier, such as alveolar edema and hyaline membrane or in lungs showing heterogeneous morphological progression, where relatively uninvolved areas were interspersed among more advanced lesions.

These findings suggest that there is a decrease of SARS-CoV replication and, consequently, a decrease in intracellular viral copy numbers in lung tissue after the first two weeks of disease, with the terminal event not dependent on continued widespread viral replication. It has also been noted that viral loads detected by RT-PCR or viral culture from nasopharyngeal aspirates, urine, and stool samples start to decrease 10–15 d after symptom onset, correlating with the time taken for the development of specific anti-SARS antibodies, which starts approximately 10 d from disease onset [5,20].

The identification of the cells infected by SARS-CoV in humans has yielded contradictory findings. To et al., using ISH combined with IHC, found dual labeling of only epithelial cells using AE1/AE3 and negative staining for macrophages (antibody CD68) [21]. In contrast, Chen and Hsiao, using ISH, demonstrated a positive signal in carbon-containing macrophages and also within vascular lumina [22]. A later report, also from Taiwan and using ISH, demonstrated positive signal in pneumocytes and not in macrophages, but it is not clear if the same patient material (from a 36-year-old woman) was used in these last two publications [23]. The discrepancies between these findings may be partially explained by three factors: the choice of probes, the age of the patient, and the disease progression. To et al. [21] used the membrane (M) gene, whilst Chow et al. [23] used a mixture of probes (N, M, and replicase [REP]) and found the greatest intensity with the N and M gene and minimal staining with the REP. Our findings support those of both To et al. and Chow et al., in that there was mainly epithelial staining but also definite macrophage (CD68) dual staining, suggesting that, although epithelial cells are the main target of SARS-CoV, the presence of viral RNA in macrophages represents either phagocytosis or low-level replication in these cells. None of these previous ISH-based studies have demonstrated ISH-positive lymphocytes, as reported by Gu et al. [24].

Our data also suggest that pneumocytes may be infected first, followed by macrophages; TO case 1, who died 5 d after symptom onset, had staining of pneumocytes but no other cells, whereas all other cases, who died five or more days after symptom onset had both pneumocyte and macrophage staining. It is possible that the material used for the To et al. study came from early cases. Shieh et al. describe finding type 2 pneumocytes positive for SARS-CoV in one patient [15]. However, in this study, staining appeared to be restricted to morphological type 1 pneumocytes, in keeping with the findings of Haagmans et al. [25]. However, Haagmans et al. were unable to demonstrate macrophage uptake of SARS-CoV [25]. We also found occasional bronchiolar epithelial cells that were positive for SARS-CoV by ISH, IHC, and electron microscopy, which has not been noted in other studies. To this extent, the immunohistochemical findings are similar to those seen with respiratory syncytial virus (RSV) infections, in which there is staining of bronchial epithelial cells, pneumocytes, and macrophages [26]. The age of the patient is also important, as SARS has been associated with greater mortality in the elderly than in the younger age group, and laboratory studies have shown that “aged” mice infected with SARS-CoV had more severe disease and greater cytokine production than younger mice [27]. Thus, differences in age may also be responsible for some of the differences noted between published results.

The spectrum of pulmonary lesions in the cases described in this study is characteristic of the acute and resolving stages of acute lung injuries, including DAD due to intrapulmonary or extrapulmonary causes such as bacterial and viral infection, trauma, shock, etc. Unlike the findings of Gu et al., who demonstrated ISH-positive cells in giant cells [24], we repeatedly found no evidence of positive staining by ISH or IHC in the giant cells in our material. As giant cells were seen mainly in patients after 14 d of disease duration, whether they have the same mechanism of formation as seen in animal models needs further investigation. Interestingly, multinucleated epithelial cells have been noted in lung infections due to other viruses such as measles, parainfluenza, and respiratory syncytial viruses. In fact, none of the changes observed in the present cases is unique for SARS-CoV infection. The pathophysiological mechanisms underlying DAD and subsequent pulmonary fibrosis have been investigated in clinical and experimental models [28,29]. It has been shown that proinflammatory and fibrogenic cytokine pathways are activated within the first 24–48 h following pulmonary insult [30,31]. High initial levels of these activities are associated with persistent pulmonary damage and increased risks of subsequent pulmonary fibrosis and poor outcome in DAD [32–34].

Previous studies have published viral loads per gram of tissue using quantitative PCR. This quantitative PCR method has been shown to be of value in determining viral load of viruses in peripheral blood, such as Epstein-Barr virus; but unlike blood, lung tissue is not homogeneous and, in an assessment of viral copies per gram of tissue, whether the tissue sample contains bronchus, scar tissue, edema fluid, or exudates is crucial. In addition, blood in tissues may lead to false-positive PCR results due to viremia rather than viral replication in the tissue. In this study, persons with high SARS-CoV viral loads in their lung tissue had good correlation with the IHC and ISH results [18], but lower lung and organ tissue viral loads showed negative results. These lower viral loads may reflect a small amount of residual genome from a previous infection comparable to RSV infections reported in both stable as well as acute exacerbation of chronic obstructive pulmonary disease patients [35]. This is a possible explanation for the low level of RT-PCR positivity in the lung tissues and in other tissues in SARS patients in which we could not detect SARS-CoV by IHC or ISH. Furthermore, in the fatal cases of SARS most patients had seroconverted at the time of death (unpublished data). However, we cannot exclude the possibility of low-level replication in these tissues that was not detectable by IHC and ISH, due to possible limitations of the sensitivity of these assays.

This study has shown that alveolar epithelium and macrophages are the chief targets of SARS-CoV infection in the human lung, in agreement with the findings reported in a primate model of the disease [10]. The predominant infiltrating cell in the lungs were macrophages, and this agrees with finding of high levels of macrophage-trophic chemokines in vivo, and in response to SARS-CoV infection in vitro [36]. However, the relative contributions of direct viral damage versus immunopathology in the pathogenesis of SARS remains unclear. Detectable levels of intracellular viral RNA and proteins in lung tissue are present in only the earliest phase of the disease, and they disappear by 2 wk after the onset of symptoms. Although we found only four positive cases by ISH and IHC, and all these were in patients who died within the first 2-wk period, it is likely that many of the negative postmortem cases would have been positive had a lung biopsy been performed earlier. Lack of IHC and ISH staining of extrapulmonary tissues in fatal cases of early SARS suggest that viral involvement of major organs may not be as widespread as data based on RT-PCR results have previously suggested. Our findings have important implications on the clinical and therapeutic management of SARS should it return in the future, in that if antiviral therapy is to be instituted, there may be a broader window of opportunity for intervention with antiviral therapy than there is, for example, with influenza, in which treatment within 48 h is required for discernible clinical impact.

**Patient Summary**

**Background**

The first SARS (severe acute respiratory syndrome) outbreak started in November 2002 in China, and over the next five months spread to a number of other cities around the world. During that time, 20%–30% of the infected people became seriously ill, and 10% died. SARS is caused by the SARS-CoV, which infects the lungs of patients and leads to breathing problems. Since the outbreak, scientists around the world have been working hard to understand the SARS virus and how it causes disease, and to develop drug treatments and vaccines.

**Why Was This Study Done?**

For this study, researchers from Hong Kong and Toronto (both cities hit by the SARS outbreak) joined forces for a large and detailed study of the bodies of patients who had died from SARS. They hoped that this would help them to understand how the virus had killed the patients, and when and how certain treatments might be most effective. The researchers wanted to see whether there were differences between individuals who died from SARS at different times after they first fell ill. They were also interested in the distribution of SARS-infected cells in the lungs of these patients, and whether they could detect the SARS virus outside of the lung in other parts of the body.

**What Did the Researchers Do and Find?**

They studied the organs of 32 patients who had died from SARS. The researchers had detailed clinical information on each of them. Some had died less than a week after they became ill, and others had died after more than a month. The researchers used a variety of modern scientific tools to visualize exactly where in the bodies the SARS virus had reached. They detected the virus in the lungs of most patients who died within two weeks of falling ill, but not in the lungs of any of the patients who died after more than two weeks of illness. Where detectable, the virus was mostly found in an area of the lung called the alveolar epithelium. Each of your lungs is made up of a network of breathing tubes, and each tube ends in a tiny pouch called an alveolus, which is surrounded by blood vessels. When you breathe in oxygen, it eventually passes into your bloodstream across the lining of these pouches, and this lining is the alveolar epithelium. The researchers also examined other organs (including heart, kidney, and liver) from two of the patients whose lungs had tested positive for the virus and three others who had shown no sign of virus in the lungs. They did not detect the virus in any of these samples.

**What Does This Mean?**

These results suggest that the SARS virus causes illness and death by attacking cells in the alveolar epithelium. The virus multiplies mostly within this group of cells, and only for a limited time after infection. Antiviral drugs are likely to be only useful during this initial time window after infection. After less than two weeks, the body's immune system seems to be able to fight back and prevent the virus from further multiplying, but this does not lead to recovery in all patients. In fact, most of the patients in this study (25 out of 32) died more than two weeks after they became ill and without signs that the SARS virus was still multiplying in their body. This suggests that in patients who die, the initial damage to the lungs is so severe that they cannot recover, and this is not dependent on continued virus replication. The study also suggests that death from SARS is not due to the virus multiplying outside of the lungs.

**Where Can I Find More Information Online?**

The following Web sites provide information on SARS.

Pages from the Government of Hong Kong:

http://www.info.gov.hk/info/sars/eindex.htm

Health Canada pages:

http://www.phac-aspc.gc.ca/sars-sras/index.html

Pages from the US Centers for Disease Control and Prevention:

http://www.cdc.gov/ncidod/sars/

World Health Organization pages:

http://www.who.int/csr/sars/en/

MedlinePlus pages:

http://www.nlm.nih.gov/medlineplus/severeacuterespiratorysyndrome.html

## Abbreviations

CoV: coronavirus
DAD: diffuse alveolar damage
IHC: immunohistochemistry
ISH: in situ hybridization
SARS: severe acute respiratory syndrome
SARS-CoV: SARS coronavirus
